# Novel halo- and thermo-tolerant *Cohnella* sp. A01 L-glutaminase: heterologous expression and biochemical characterization

**DOI:** 10.1038/s41598-019-55587-9

**Published:** 2019-12-13

**Authors:** Samaneh Mosallatpour, Saeed Aminzadeh, Mehdi Shamsara, Reza Hajihosseini

**Affiliations:** 1 Bioprocess Engineering Group, Institute of Industrial and Environmental Biotechnology, National Institute of Genetic Engineering and Biotechnology (NIGEB), Tehran, Iran; 2 Faculty of Science, Payame Noor University, Tehran, Iran; 3 Animal Biotechnology Department, Institute of Agricultural Biotechnology, National Institute of Genetic Engineering and Biotechnology (NIGEB), Tehran, Iran

**Keywords:** Enzymes, Hydrolases, Biochemistry, Biotechnology, Molecular biology

## Abstract

L-glutaminase importance to use in the food industry and medicine has attracted much attention. Enzymes stability has always been a challenge while working with them. We heterologously expressed and characterized a novel stable L-glutaminase from an extremophile bacterium (*Cohnella* sp. A01, PTCC No: 1921). K_m_, V_max_, catalytic efficiency and specific activity of rSAM were respectively 1.8 mM, 49 µmol/min, 1851 1/(S.mM) and 9.2 IU/mg. Activation energy for substrate to product conversion and irreversible thermo-inactivation were respectively 4 kJ/mol and 105 kJ/mol from the linear Arrhenius plot. rSAM had the highest activity at temperature 50 °C, pH 8 and was resistant to a wide range of temperature and pH. In compare to the other characterized glutaminases, rSAM was the most resistant to NaCl. Mg^2+^, glycerol, DTT, and BME enhanced the enzyme activity and iodoacetate and iodoacetamide inhibited it. rSAM had only been partially digested by some proteases. According to the Fluorimetry and Circular dichroism analysis, rSAM in pH range from 4 to 11 and temperatures up to 60 °C had structural stability. A cysteine residue in the enzyme active site and a thiol bond were predicted upon the modeled tertiary structure of rSAM. Present structural studies also confirmed the presence of a thiol bond in its structure.

## Introduction

Enzymes derived from microorganisms particularly from extremophiles due to their sustainability are appealing for chemical processes designing for many industrial and medical purposes^1,2^. L-glutaminase (EC. 3. 5. 1. 2) hydrolyzes the amide bond in the L-glutamine side chain and produce L-glutamate and amine and can be found in almost all creatures. This enzyme supplies the required nitrogen and carbon source of the biosynthesis of several intermediates in metabolic pathways such as DNA structural units and amino acids, therefore plays a crucial role in cellular metabolism. L-glutaminase with its proven abilities to use in the food industry and cancer treatment has become more important in the last few decades^3–5^. The other L-glutaminase applications are in culture medium as glutamine biosensor^6,7^, in GABA^8^ and theanine synthesis^9^ and also to measure the reaction rate of threonine synthesis^10^. L-glutaminase by releasing L-glutamate as a flavor enhancer amino acid from L-glutamine can be used in the food industry^10,11^. One of the foodstuff production processes in which this enzyme is widely used is soy sauce manufacturing. Soy sauce in Asian countries is one of the most desired seasonings and its production includes two-steps fermentation named “koji and moromi fermentation”. At these steps this enzyme has been found to be the intermediary that by produce L-glutamate from L-glutamine originated from soybean protein, improves the soy sauce flavor and gives it so-called “umami” taste. During the moromi fermentation pH level is reduced to nearly 4–5 and applying the higher temperature of 45 °C along the production causes longevity of the soy sauce. During the fermentation at high temperatures, low concentration of ethanol is also produced. Presence of high amounts of salt (14–20%) is a common condition in the soy sauce fermentation thus L-glutaminases which are capable to withstand low pH, high temperature and the presence of NaCl and ethanol are preferred to use for soy sauce production^12,13^.

To tackle these problems and due to the importance of the enzymes with appreciable activity in harsh conditions which is the scientific world concerns, in the present research after heterologous expression of L-glutaminase from an indigenous thermophilic bacteria (*Cohnella* sp. A01 PTCC No 1921) and full activity and structural characterization under different conditions, rSAM was found to have high resistance and rigid structure.

## Results

### SAM heterologous expression and purification

**Figure 1:**
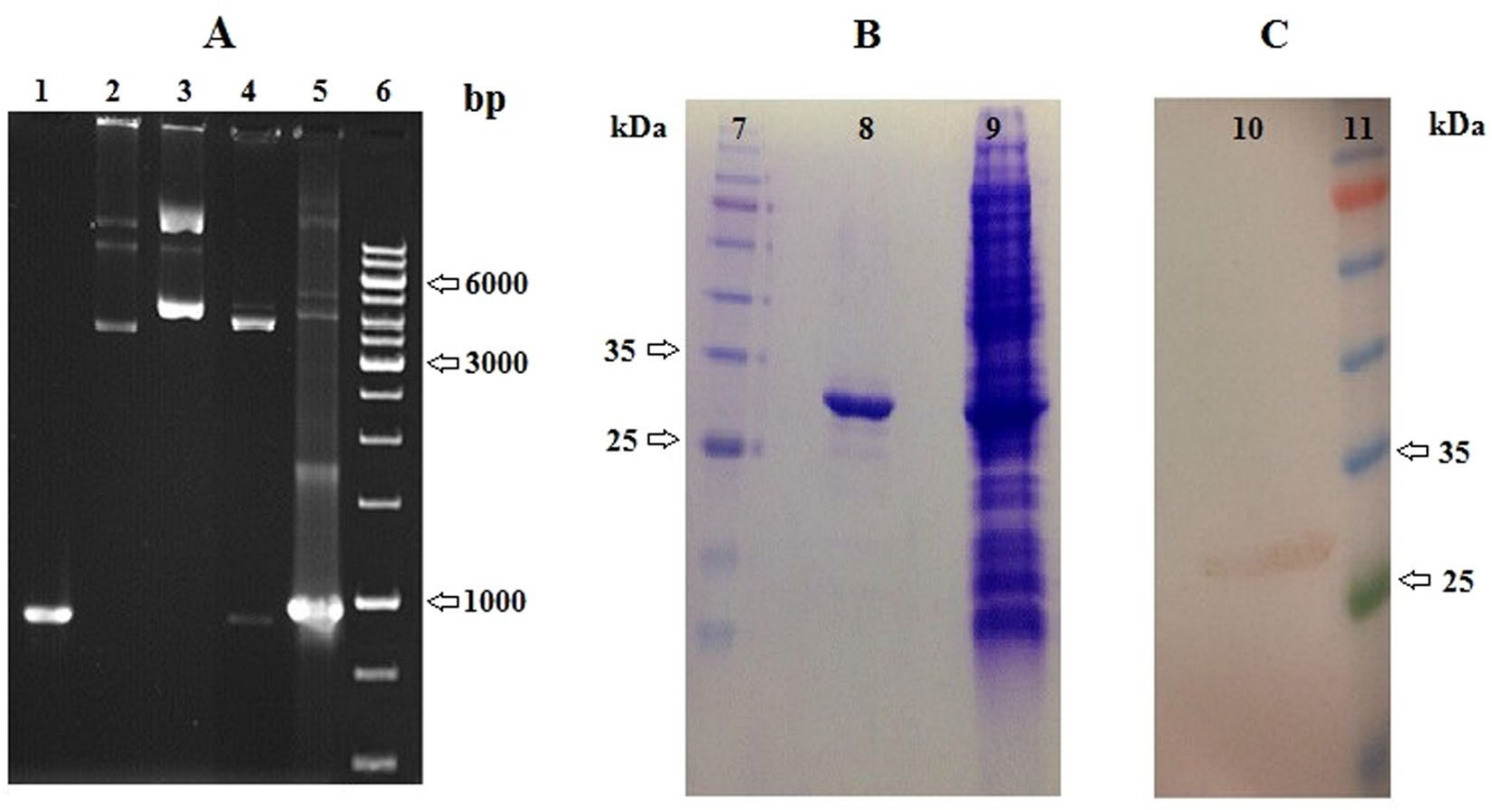
Cloned (**A**) and expressed (**B,C**) *Coh* 03501 gene. Lane 1: PCR product. Lane 2: non-recombinant pET26b. Lane 3: recombinant pET26b. Lane 4: digested pET26b with *Nde* I and *Not* I. Lane 5: colony PCR, Lane 6: DNA ladder, Lane 7: protein molecular mass marker. Lane 8: purified rSAM (protein concentration: 0.4 mg/ml), Lane 9: total cellular expressed protein from *E. coli* BL21 (DE3) (protein concentration: 1.6 mg/ml). Lane 10: a single band from the western blotting analysis against the rSAM His-tag sequence. Lane 11: protein molecular mass marker.

Cloning was verified by the movement difference between pET-26b and rpET-26b (Fig. 1A, lane 2 and 3) and also between digested rpET-26b and colony PCR (Fig. 1A, lane 4 and 5). The single band of rSAM after purification with molecular weight of 34 kDa was shown in Fig. 1B lane 8 and the total cellular expressed protein from *E. coli* BL21 (DE3) was shown in Fig. 1B lane 9. Western blotting against the His-tag sequence of rSAM confirmed the enzyme heterologous expression (Fig. 1C, lane 10). Sequencing was carried out and the complete nucleotide sequence of rSAM gene was submitted to the GenBank database, under the GenBank accession number: MH973594 and the protein ID is QCI03326.1.

### Enzyme qualitative assay

rSAM qualitative assay on Agar-phenol red plate which had L-glutamine as enzyme substrate showed changing in medium color surrounding the enzyme-containing well from yellow to red (Supplementary Fig S1A-well 3). Around the control wells (Supplementary Fig S1A-wells 1&2) and also on the Agar-phenol red plate that had no L-glutamine (Supplementary Fig S1B) no color changes found.

### Substrate specificity

Specific substrate investigating showed that at a fixed concentration of amide bond-containing chemicals as the substrate, rSAM was found to have no activity in the presence of tested molecules except L-glutamine.

### Effect of temperature and pH on the rSAM activity and stability

**Figure 2:**
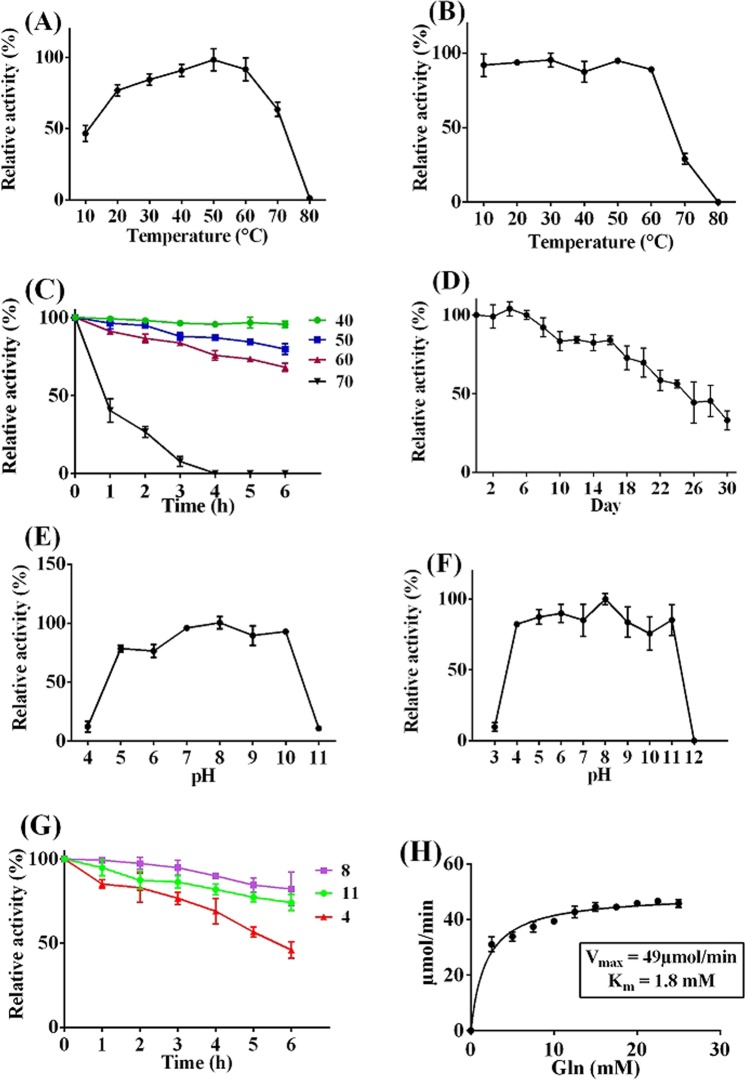
rSAM temperature and pH characteristics. (**A**) Temperature profile demonstrating the rSAM optimum temperature at 50 °C. Temperature stability at: (**B**) 10–90 °C in 90 min, (**C**) 40, 50, 60 and 70 °C in different times, (**D**) 25 °C in different days up to a month. (**E**) pH profile illustrating the rSAM maximum activity at pH 8. (**F**) rSAM pH stability at pHs 3–12 in 90 min showing the rSAM stability at a wide range of pH (4–11). (**G**) pH stability at pHs 4, 8, 11 in different times demonstrating the rSAM resistance at mentioned pH up to 6 hours more than 50% of relative activity. (**H**) rSAM Michaelis-Menten plot. Each value represents the mean ± SD for three determinations. The software used was “GraphPad prism 6”, URL: https://www.graphpad.com/scientific-software/prism/.

Temperature profile performance of the rSAM illustrated that the enzyme maximum activity was at 50 °C and approximately a gradual reducing with a gentle slope was observed over the upper and lower temperatures (Fig. 2A). In the other words the optimum temperature of rSAM was 50 °C and at temperatures near that had a significant activity.

Evaluation of the enzyme temperature stability at different temperatures for different lengths of time showed that the rSAM was active higher than 80% of activity at temperatures between 10–60 °C for 90 min and lost its activity at temperatures above 70 °C at the end of 90 min (Fig. 2B). As shown in Fig. 2C calculation of the rSAM residual activity following the incubation at 40 to 70 °C for 1–6 hour, demonstrated that at 40 °C rSAM has kept its activity almost above 95% and at 50 and 60 °C by increasing the incubation time, enzyme activity has reached to nearly 80% and 70% respectively at the end of 6 hours. At 70 °C, about 40% activity after 1 hour was observed.

Above all, after 26 days of rSAM incubation at 25 °C for 30 days, only half of the enzyme activity was lost, so the rSAM half-life at room temperature was 26 days (Fig. 2D). Altogether, Fig. 2A–D show the high rSAM thermal stability.

rSAM maximum activity was found at pH 8 however at pHs 7, 9 and 10 exhibited more than 90% of maximum activity. rSAM activity was slightly decreased at pHs 5 and 6 in such a way that only 10–15% of enzyme activity has declined compared to the pHs 7, 9 and 10 (Fig. 2E).

Results of the pH stability in 90 min (Fig. 2F) revealed that the enzyme was stable at pHs between 4–11 more than 80%. Enzyme exposing to the pHs 5, 8 and 11 and examine its activity every hour up to 6 hours showed that rSAM activity at optimum pH 8 until 3 hours retained almost 95% and over the time, its activity decreased with a very mild slope until the end of 6 hours. At pHs 5 and 11 enzyme activity reduced more than at optimum pH but enzyme stability at pH 11 was less than at pH 5 and after 5 hours reached slightly below the 50% (Fig. 2G).

### Influence of various reagents and chemicals on the rSAM activity

Exploration of the various metal ions effect on the rSAM activity at final concentrations of 25 and 125 mM revealed that in the presence of MgCl_2_ enzyme activity enhanced approximately two and half times. rSAM also retained its high activity in the presence of CoCl_2_ 25 mM. ZnCl_2_ 125 mM, FeCl_3_ 25 mM and ZnSO_4_ 125 mM significantly decreased the enzyme activity and some salts including CuSO_4_, AgNO_3_, and FeCl_3_ 125 mM completely ceased rSAM (Table 1).

**Table 1:** The effect of different final concentration of some metal ions, reducing and thiol binding agents, detergents, metal chelator and chemical solvents on the purified rSAM relative activity.

Modulator	Relative activity (%)			Modulator	Relative activity (%)		
Metal ions				Thiol blocking agents			
Control	100 ± 3.92			Control	100 ± 4.7		
**Concentration (mM)**	**25**	**125**		**Concentration (mM)**	**2.5**		**5**
Mg^2+^	241 ± 4	217.1 ± 10.5		Iodoacetamide	68 ± 12.2		40.61 ± 3
Co^2+^	234 ± 3	107.8 ± 8.7		Iodoacetate	74.2 ± 2.2		52.7 ± 4
Zn^2+^	110 ± 3.5	11.3 ± 0.9		**Detergents**			
Li^2+^	101.5 ± 6	96.3 ± 5.6		Control	100 ± 2		
Ni^2+^	67.1 ± 6	26.7 ± 4		**Concentration (%v/v)**	**1**	**2**	**5**
Ca^2+^	106 ± 8.6	93.1 ± 10.1		Tween 20	57 ± 7	64 ± 8	11.6 ± 5
Ba^2+^	109 ± 6.6	0		Tween 80	54 ± 9.6	32 ± 1	0.9 ± 1
Fe^2+^	128.3 ± 1	112.5 ± 5.3		Triton 100-X	22.5 ± 3	13 ± 5	1.7 ± 0.2
Fe^3+^	12.1 ± 0.8	0		SDS	47.4 ± 5	0	—
Al^3+^	46.6 ± 7.4	16.6 ± 3.3		**Chemical solvents**			
K^+^	113.8 ± 3	91.1 ± 3.1		Control	100 ± 2.1		
ZnSO_4_	94.7 ± 8	10.9 ± 2.6		**Concentration (%v/v)**	**5**	**15**	**25**
CuSO_4_	0	0		Ethanol	86.2 ± 5.2	81.5 ± 3	28 ± 2
AgNO_3_	0	0		Methanol	99.8 ± 5	74 ± 11	85.5 ± 6
**Reducing agents (mM)**				Isopropanol	86.7 ± 5.2	82 ± 8	37 ± 4
Control	100 ± 2.2			Acetone	27.9 ± 8.5	40 ± 7	0
BME(5,10, 30)	126.2 ± 10	186 ± 3	286 ± 2	Glycerol (5, 25 & 33 %)	102.2 ± 2.4	105 ± 4 (25% glycerol)	124 ± 7 (33% glycerol)
DTT(2.5,5, 20)	106. ± 6	132 ± 2	143 ± 1	**Metal chelator**			
**NaCl Concentration (% w/v)**	**Specific activity (U/mg)**			Control	100 ± 4		
Control	9.2			**Concentration (% w/v)**	**5**		**25**
5	9.9			EDTA	74.1 ± 0.8		56 ± 1
10	10.13						
15	11.04						
20	10.8						
25	8.99						

As shown in Table 1 by enhancing the BME and DTT concentrations, enzyme activity increased. By assessment the impression of chemical solvents including ethanol, methanol, isopropanol, acetone and glycerol on the enzyme activity, was proven that ethanol and isopropanol at a final concentration of 25% and acetone at all examined concentrations diminished the enzyme activity nearly 50%. rSAM could perform its activity without much changes in the presence of ethanol, methanol, isopropanol at final concentrations of 5, 10% and even glycerol had an increasing effect on the rSAM activity specially accelerated the enzyme activity at final concentration of 33%. Iodoacetamide and Iodoacetate as thiol blocking agents, Tween 20, Tween 80, and Triton 100-X, SDS as detergents and EDTA caused reduction in enzyme activity at all tested concentrations.

### rSAM NaCl tolerance

Compared to the control without NaCl (Supplementary Fig. S2A), by increasing the NaCl concentration up to 20% (w/v), rSAM remained active with a gradual increase in activity. Addition of 25% (w/v) NaCl to the reaction reduced the enzyme activity slightly (Supplementary Fig. S2B), so that at 25% (w/v) of NaCl, rSAM was still active more than 90%. The quantitative values of the rSAM specific activity in presence of mentioned concentration of NaCl were shown in Table 1.

### Enzyme resistance against proteases

**Figure 3:**
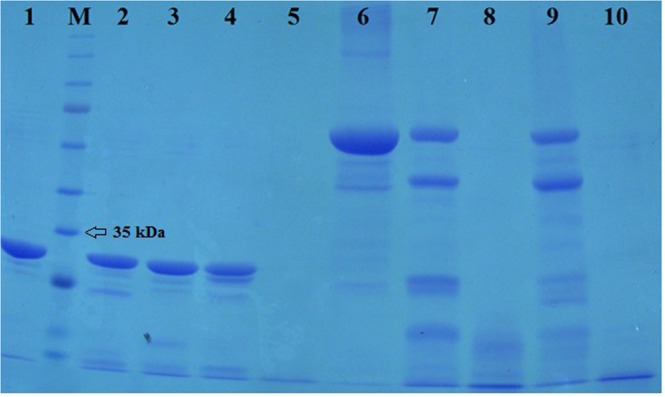
SDS-PAGE analysis of rSAM resistance against some proteases. Lane 1: purified rSAM. Lane M, protein molecular mass marker. Lane 2: rSAM - Chymotrypsin. Lane 3: rSAM - Pepsin. Lane 4: rSAM - Trypsin. Lane 5, rSAM – Proteinase K. Lane 6, Bovine serum albumin (BSA). Lane 7: BSA – Chymotrypsin. Lane 8: BSA – Pepsin. Lane 9: BSA – Trypsin. Lane 10: BSA - Proteinase K.

SDS-PAGE analysis of the rSAM and BSA digestion assay showed that despite being the large number of cleavage sites on rSAM, it approximately remained intact with very slight cuts by chymotrypsin, pepsin and trypsin but rSAM was quite hydrolyzed by proteinase K. Compared to the rSAM, BSA as a control protein is broken down to the large extent by chymotrypsin and trypsin and entirely destroyed by pepsin and proteinase K (Fig. 3).

### rSAM Kinetic and thermodynamic parameters

As regards the Michaelis-Menten plot shown in Fig. 2H, rSAM Michaelis constant (K_m_) and maximum velocity (V_max_) were calculated to be 1.8 mM and 49 µmol/min respectively and the catalytic efficiency and specific activity were calculated to be 1851 1/S.mM and 9.2 IU/mg respectively. Supplementary data Fig. S3A shows the Arrhenius plot for rSAM temperature activation up to 50 °C. The rSAM activation energy from the Arrhenius plot was calculated to be 4 kJ/mol which is the amount of energy that must be provided for rSAM to catalyze the L-glutamine-L-glutamate conversion. rSAM residual activity over the time at various temperatures and its irreversible heat inactivation Arrhenius plot were respectively shown in Supplementary Fig. S3B,C. Deduced thermodynamic parameters from the Arrhenius plot based on the rSAM temperature profile and stability and the enzyme half-life at temperatures 40, 50, 60 and 70 °C are shown in the Supplementary Table S1.

### Structural studies

#### Fluorescence analysis

**Figure 4:**
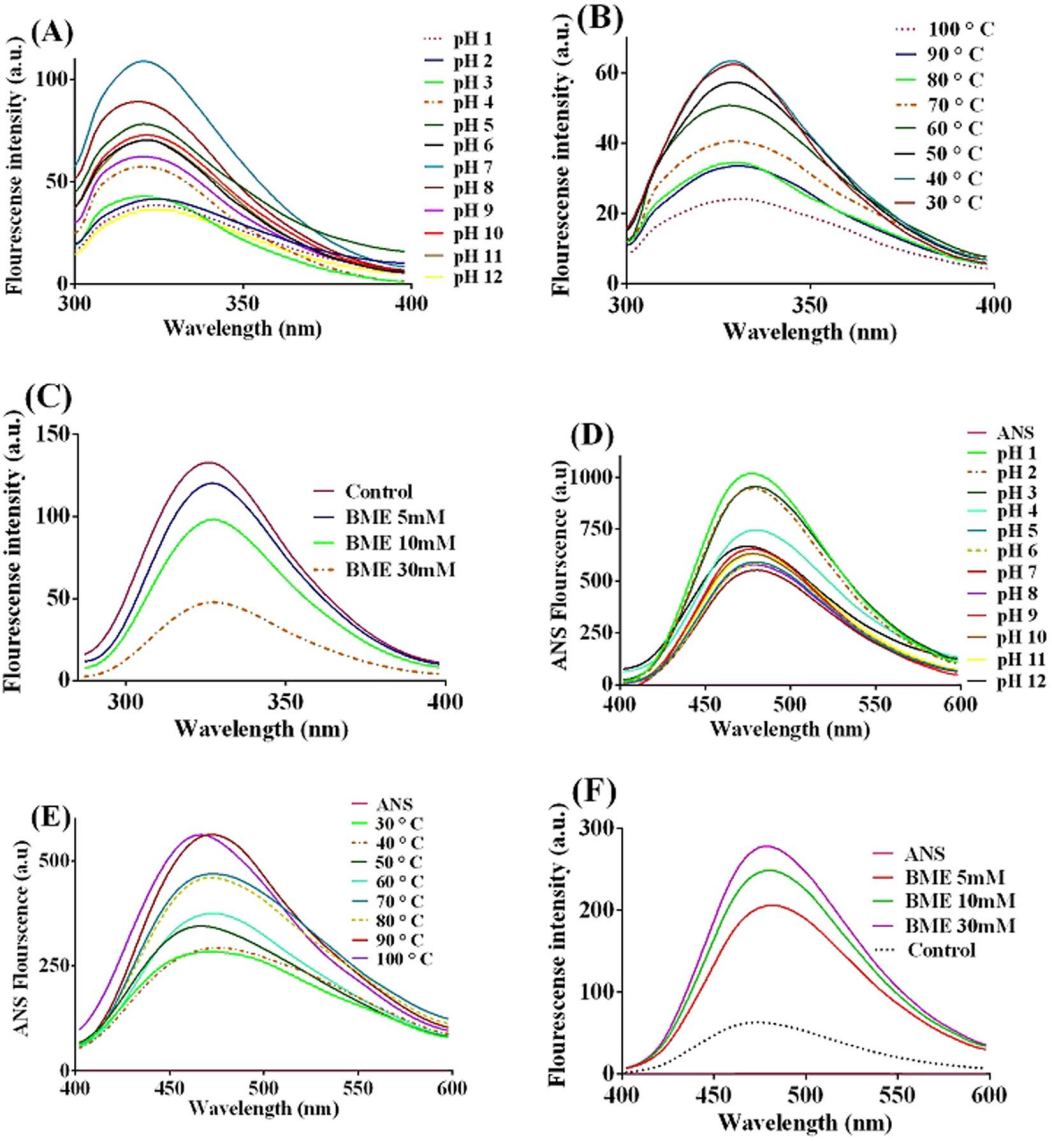
rSAM intrinsic (**A–C**) and extrinsic (**D–F**) fluorescence spectroscopy: (**A**) at different pHs (1–12): maximum emission was at pH 7, at pH 8 which is the optimum rSAM pH intrinsic emission was reduced, (**B**) at various temperatures (30–100 °C): at optimum temperature (50 °C) decreasing in intrinsic emission was observed compare to the 30 and 40 °C, (**C**) in the presence of 5, 10 and 30 mM final concentrations of BME: by increasing the concentration of BME, intrinsic emission was reduced, (**D**) ANS fluorescence intensity at different pHs (1–12): at pHs between 4–11 a relative stability in extrinsic emission was observed and the least amount of emission was at pH 7, (**E**) at various temperatures (30–100 °C): at rSAM´s optimum temperature (50 °C) extrinsic emission was relatively more than lower temperatures, and (F) in the presence of 5, 10 and 30 mM final concentrations of BME: by increasing the concentration of BME, extrinsic emission was enhance. The software used was “GraphPad prism 6”, URL: https://www.graphpad.com/scientific-software/prism/.

Tryptophan intrinsic fluorescence examination at various pHs (Fig. 4A) showed that at very acidic (1, 2 & 3) and alkaline (12) pHs, recombinant protein had the least amounts of emission and in addition a blue shift about 5 nm was observed at pH 3 compared to pH 1, 2 and 12. rSAM had the maximum emission at pH 7 which was somewhat more than that of at pH 8 in which rSAM had the maximum activity. In the other words, as is clear in Fig. 4A, at the neutral pH (7) the rSAM structure was more intact and by reducing and enhancing the pH, its structure altered and tryptophan residue was more exposed.

Intrinsic measurements to assess the effect of different temperatures on the protein structure demonstrated that the enzyme at temperatures 30 and 40 °C had the maximum intensity and a little reduction was observed at 50° C which was the rSAM optimum temperature. There was not much difference between the emission at 50 and 60 °C. From 80 °C the intrinsic fluorescence was significantly decreased (Fig. 4B). Thus Fig. 4B shows that rSAM at temperatures 30 and 40 °C had the relative structural stability and at optimum temperature this structural stability has been somewhat loosened and at 80 °C and upper rSAM structure was more exposed.

As shown in Fig. 4C, by increasing the BME concentration, the fluorescence intensity was reduced, while control (the sample with no BME) had the maximum spectrum emission. So Fig. 4C illustrates that the presence of BME has caused more exposure of tryptophan residue and hence more reducing rSAM rigidity.

Extrinsic fluorescence was performed to identify the binding of ANS to the exposed hydrophobic surfaces of the unfolded or partially folded state of rSAM. As shown in Fig. 4D, the control (sample without protein) had no emission that it means that ANS without no binding to the hydrophobic surfaces of protein have had no emission. rSAM at pH 7 had the minimum emission and at pH 8 which is rSAM optimum pH had a little increasing in its extrinsic emission. It means ANS at pH 7 compare to the other pHs have had the least binding to the rSAM hydrophobic surfaces and at pH 8 ANS have bonded slightly more. The highest extrinsic fluorescence was observed at pHs 1, 2 and 3 and totally at a range of 4–11 there was no significant changes in the intensity of extrinsic fluorescence. Generally, Fig. 4D clearly shows the relative rSAM structural stability at pHs 4 to 11.

Extrinsic fluorescence surveying at temperature range of 30–100 °C (Fig. 4E) revealed that the protein at 30 and 40 °C had the least ANS fluorescence and at 50 °C (rSAM optimum temperature) the fluorescence emission increased slightly. Therefore, as illustrated in Fig. 4E, rSAM at its optimum temperature have had not much rigidity. Until 60 °C considerable changes in the fluorescence intensity was not observed. Extrinsic fluorescence test was also approved that the higher the concentration of BME, the greater the ANS binding to the hydrophobic surfaces (Fig. 4F). So ANS binding to the hydrophobic surfaces in Fig. 4F was also confirmed the effect of BME on the rSAM structure.

Quenching effect of the acrylamide and rSAM Stern-Volmer plots at pHs 4, 8 & 11, temperature range of 40–70 °C and also in the presence of BME at final concentrations of 5, 10 and 30 mM were shown in Supplementary Fig. S4. Comparison of the A, B and C parts of Supplementary Fig. S4 showed that the rSAM structure at pHs 4 and 11 was more changed than at pH 8. In such away the lower effect of acrylamide in fluorescence quenching was observed at pH 8 compared to the pHs 4 and 11. The Stern-Volmer plots showed in Supplementary Fig. S4,D was also illustrated the lower Quenching effect of acrylamide on the tryptophan emission at pH 8 than at pHs 4 and 11. Quenching effect of the acrylamide at temperatures 40–70 °C (Supplementary Fig. S4,E–I) showed the lowest effect at temperature 40 °C and at temperature 50 and 60 °C there was not much differences and at temperature 70 °C acrylamide quenching was very faster at lower concentration (100 mM). Corresponding Stern-Volmer constants (K_sv_) were also calculated (Supplementary Table S2). The Stern-Volmer constants at temperatures 40–50 °C were also not very differ. As shown in Supplementary Fig. S4,J–M the quenching of tryptophan emission and Stern-Volmer constants were increased by enhancing BME concentrations.

#### Circular dichroism analysis

**Figure 5:**
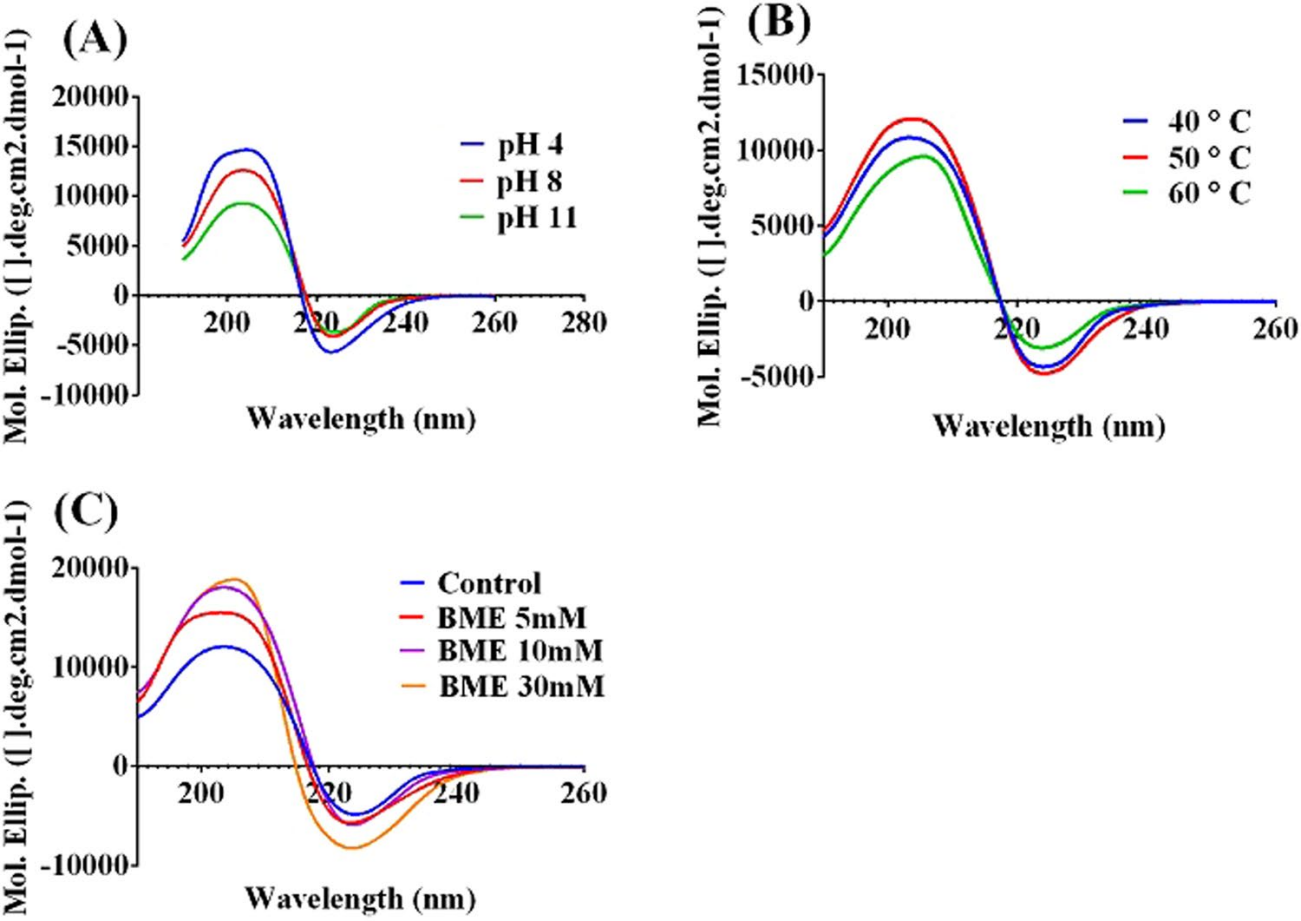
Far-UV CD spectra of rSAM at: different pHs (**A**), temperatures (**B**) and BME concentrations (**C**) were shown no much changes in the rSAM´s secondary structure. The software used was “GraphPad prism 6”, URL: https://www.graphpad.com/scientific-software/prism/.

Alterations in the secondary structure extent of the rSAM under various pHs, temperatures and BME concentrations using Far-UV CD were shown respectively in Fig. 5A–C. The Far-UV spectra and the secondary structure at rSAM optimum pH (8) and temperature (50 °C) which is included in Supplementary Table S3 showed the more β-sheets in the rSAM structure and as shown in Fig. 5A, by changing the pH, temperature and BME concentration, no major changes were found in the secondary structure.

### *In-silico* analysis

rSAM protein BLAST analysis was carried out to find similar sequences and protein Blast search showed the that the query sequence had the highest homology with Glutaminase A with GenBank: REK63647.1 with 99% identity and the phylogenetic tree was drawn phylogenetic tree by similar proteins showed the most similarity to the *Cohnella* species and paenibacillus bacteria as other members of the paenibacillaceae family. Homology protein sequences assessment with two other salt-tolerant glutaminases (*Micrococcus luteus* glutaminase and *Streptomyces flavogriseus* glutaminase) showed no much similarity and glutaminases belong to the human are in different clade (see Supplementary Fig. S5). rSAM bio-molecular 3D structure prediction and active site amino acids were shown in Supplementary Fig. S6,A. As shown in Supplementary Fig. S6B rSAM had a disulfide bond between Cys.63 and Cys. 209.

## Discussion

Considering the widespread applications of enzymes in various conditions and their high activity and specificity, there is an increasing requisiteness to explore and identify the novel ones with the specific characteristics. The environment is a great resource for diverse microorganisms and some of them are extremophile, hence there is an interest to identify such germs to isolate their enzymes which are able to resist tough conditions^14^. Since L-glutaminase is one of the enzymes that has potentials to apply in both industry and medicine, in the present study we heterologously expressed the L-glutaminase coding gene from a new indigenous thermophilic bacterium, *Cohnella* sp. A01, PTCC No 1921, and evaluation of its functional, kinetic and structural characterizes was carried out. *Cohnella* sp. A01 was isolated from the water and waste water of Abadan shrimp farming ponds in southwestern of Iran and by the 16 S rRNA gene study and biochemical and microbiological analyses with the Bergey’s Manual of Systematic Bacteriology, the A01 strain was classified as a bacteria belonging to the genus *Cohnella*. Subsequent sequence analysis of the 16 S rDNA gene confirmed the isolate as being *Cohnella* sp and the nucleotide sequence of 16 S rDNA from *Cohnella* sp. A01 would be made available in the GenBank nucleotide sequence databases (accession number: JN208862)^15^.

The purified rSAM had molecular weight nearly 34 kDa and in compared to the L-glutaminases from the most other microorganisms is approximately lower and this issue makes the putative enzyme more suitable for using in pharmacy industry as a therapeutic enzyme compared to the others (see Supplementary Table S4). The molecular weight of obtained L-glutaminases from several microorganisms are very different which indicates that L-glutaminase molecular mass depends on the type of living creature.

Except the quantitative assay (Nesslerization) to determine the enzyme total activity, red color halo appearance around the rSAM-containing well on the agar-phenol red plate as the qualitative assay is due to the liberated ammonia from L-glutamine by rSAM and means that its activity is to the extent that can produce enough ammonia from a defined concentration of L-glutamine and enhance the medium pH. Some L-glutaminases were not very specific toward their natural substrate (L-glutamine) and could hydrolyze the amide bond in different molecules. Substrate specificity analysis revealed that the rSAM deaminated only L-glutamine and had no activity in the presence of any other examined molecules. In the other words, rSAM is very specific toward its natural substrate whereas as shown in Supplementary Table S4 the most other L-glutaminases from other microorganisms which have been characterized until now, not only hydrolyzed the amide bond in L-glutamine, but also have had the amide bond hydrolysis feature on the other amid bond-containing molecules as substrate. Supplementary Table S4 illustrates that only L-glutaminases from *Bucillus subtilis* (YbgJ, Yb1M) and *E. coli* (YneH, YbaS)^16^ like rSAM have the strict substrate specificity toward L-glutamine.

rSAM maximum activity was at 50 °C. The optimum temperature of glutaminases from *Bacillus sp*. LKG-01 (MTCC 10401)^17^, *Stenotrophomonas maltophilia* NYW-81^13^ and *Bacillus amyloliquefaciens*^18^ are respectively at 70, 60 and 60 °C and *Aspergillus sojae*^19^ and *Micrococcus luteus* K-3^20^ glutaminases like rSAM have the most activity at 50 °C and the rest of glutaminases have lower and different optimum temperature (see Supplementary Table S4). As a result, various glutaminases have different optimum temperature and rSAM can be categorized in thermophilic enzymes. Enzymes isolated from thermophiles not only exhibit unusual stability to heat and protein denaturants, but also display enhanced protein rigidity in respect to that of their mesophilic counterparts. It can be proposed that thermophilic enzymes have rigid structure and an appropriate level of protein mobility is required for enzyme catalysis^21,22^. rSAM remained active and stable at a wide range of temperature 10–65 °C. As already mentioned, glutaminases in food industry are widely used and since under these conditions temperature commonly goes up. In soy sauce production temperature reaches to 45 °C, therefore, thermophilic enzymes are preferred to use in this industry. Since the rSAM half-life at room temperature is 26 days, accordingly, it can be applied with no need to be kept at low temperature for several days.

rSAM optimum pH was 8 and by comparison of all characterized glutaminases optimum pH (Supplementary Table S4), most of them are in the alkaline region, some ones are neutral and none of them have acidic optimum pH. Therefore, it can be concluded that almost all glutaminases alkalinophile. rSAM at pHs 4–11 was stable and had more than 70% activity and since in soy sauce production the processing pH reaches to 4–5, this enzyme can be used properly[10]. There is no considerable difference between the rSAM activity at neutral pH range (7–7.5) and at its optimum pH 8 and since its activity and stability at 37 °C were significant, rSAM can act well in physiological conditions (pH 7.4, temperature 37 °C).

Table 1 shows the effect of variant factors on the rSAM activity. Amongst the tested metal ions, Mg^2+^ increased the enzyme activity more than twice at both concentrations 25 and 125 mM, this result suggests Mg^2+^ as the enzyme possible cofactor. BME and DTT as the disulfide bond reducing agents^23,24^ enhanced the enzyme activity, it can be concluded that the rSAM with 5 cysteine molecules has at least one disulfide bond and BME and DTT by reducing it, weaken the protein structure rigidity and cause the more exposing enzyme active site to the substrate.

Iodoacetate and iodoacetamide as alkylating agents by binding covalently to the thiol group of cysteine irreversibly inhibit the enzymes having cysteine in their active site^25–27^. These thiol binding agents decreased the activity of the enzyme (Table 1), therefore rSAM is likely to contain cysteine residue in its active site.

Generation of low amount of ethanol (4%) and using 18–20% NaCl concentration in the soy sauce fermentation processes makes it difficult to use enzymes as the biological catalysts in this pathway^12,28^. More than 50% of the rSAM activity was maintained in the presence of up to 15% v/v concentration of ethanol, methanol and isopropanol. As shown in Table 1, rSAM specific activity in the presence of up to 20% NaCl concentration was increased and 25% salinity level reduced the enzyme specific activity slightly. The rSAM activity assay in the presence of 0–25% of NaCl were shown in Supplementary Fig. S4. Compared to the other characterized L-glutaminases (Supplementary Table S4), rSAM has the most salt tolerance and to date, no glutaminase has been reported with such salt resistance. Totally, with regards to the rSAM resistance against ethanol, a wide range of pH, temperature and high concentration of NaCl, it can be valuable in the food industry particularly soy sauce production. The application of the rSAM in the soy sauce production was patented under No. 98799 in “Real State Registration organization of Iran”.

There is no reduction in the enzyme activity in the presence of glycerol as a chemical chaperone^29^ and 33% v/v glycerol concentration enhances rSAM activity. As a result we can use glycerol to keep the enzyme at −20 °C for a long time with no negative effect on its activity.

Chymotrypsin, pepsin, trypsin and proteinase K as proteolytic enzymes have specific cleavage sites on the amino acid sequences to break down the proteins and polypeptides^30–32^. According to the Supplementary Fig. S7 elucidating the rSAM (A) and BSA (B) cleavage sites for such protease, there are so much sites for them on the rSAM amino acid sequence obtained using the translation tool at the ExPASy server (http://web.expasy.org/translate/). In spite of the existence of more than 90 cleavage sites for chymotrypsin, pepsin, trypsin and proteinase K in rSAM amino acid sequence, it was nearly showed resistance to the chymotrypsin, pepsin, trypsin and only cleaved partially by them but BSA which was used as a control protein was digested by chymotrypsin and trypsin to the large extent and completely faded by pepsin and proteinase K. Failure in rSAM digestion entirely is indicating the inaccessibility of rSAM cleavage sites and hence is reflective the rSAM rigidity as a thermophilic protein^33,34^.

For wielding the enzymes in industry and to understand the cellular organization, kinetics determination of enzymes is always has a crucial role^2^. As shown in Supplementary Table S4, the K_m_ of the glutaminases from different microorganisms are very different and rSAM K_m_ which was found to be 1.8 mM is lower than that of in many other microorganisms. This suggests the high substrate affinity of the L-glutaminase from *Cohnella sp*. A01 toward its natural substrate L-glutamine. Glutaminases from *Aspergillus oryzaezae* AJ11728^3^, *Streptomyces sp*^35^*, Rhizobium etli*^36^ have K_m_ near to the rSAM. As shown in Supplementary Table S1 the changes in activation energy (ΔE^*^), enthalpy (ΔH^*^), entropy (ΔS^*^) for rSAM thermo-activation according to the Arrhenius plot were respectively 4 kJ/mol, 1.3 kJ/mol and −0.17 J/mol°K while for L-glutaminase from *E. coli* were respectively 13.2 kcal/mol (55.44 kJ/mol), 8.2 kcal/mol (34.44 kJ/mol) and −14.1 e.u (59 J/mol°K). Calculated rSAM activation energy for the heat inactivation (ΔE^#^) was 105 kJ/mol which is near to the salt- and thermotolerant L-glutaminase from *Lactobacillus rhamnosus* (110 kJ/mol)^37^. Protein denaturation mechanism previously described^38^ acknowledges that the irreversible inactivation/denaturation of proteins/enzymes is a two-step reaction:

$${\bf{N}}\underset{{{\rm{k}}}_{-1}}{\overset{{{\rm{k}}}_{1}}{\longleftrightarrow }}{\bf{U}}\mathop{\longrightarrow }\limits^{{{\rm{k}}}_{2}}{\bf{I}}$$

N is the protein native state, U is the reversibly and partially unfolded enzyme form and I is the enzyme irreversible inactivated state. The transition state (Tn^*^) which is formed between N and U is determining the irreversible thermodynamic parameters. ΔG^#^ (58 kJ/mol) is the value usually shows the protein denaturation reaction. ΔH^#^ and ΔS^#^ are determining respectively the heat and entropy alteration in conversion reaction of native to the transition state^39^. The large amount of rSAM ΔH^#^ (102 kJ/mol) shows that the rSAM unfolding seems to be the rate-determining stage in its irreversible thermo-inactivation. Since the ΔS^#^ value is positive (0.13 kJ/mol°K) we can conclude that the rSAM unfolding in irreversible thermo-inactivation is the rate-determining step. Mostly, enzymes that have been used in industry had thermal resistance and researches have continued to find such enzymes^40^. Based on the rSAM residual activity versus heating time and the deduced half-life at temperatures 40, 50, 60 and 70 °C (see Supplementary Table S1), the enzyme is slowly deactivated by heating. The half-life of *M. guilliermondii* EM2Y61 glutaminase at 40–50 °C was nearly 12 h^28^. The half-life of glutaminases from *Bacillus sp*. LKG-01 at 50 °C^17^ and *Lactobacillus sp*. KCTC3594 at 60 °C were 30 min.^41^. *Lactobacillus rhamnosus* glutaminase had 12.9, 7.6, 3.8 and 0.9 min half-life at temperatures respectively 55, 60, 65, 70 and 75 °C^37^.

Under different conditions, proteins as biological macromolecules, depending on their structural features get different conformations^42^. rSAM intrinsic and extrinsic fluorescence at different pHs shows that the enzyme at pHs 4–11 has a relative structural stability and although the rSAM optimum pH is 8 but at pH 7 has the most structural stability. As already mentioned, thermophilic proteins isolated from thermophilic and extremophilic microorganisms mostly have a rigid structure. This protein rigidity has a significant influence on the catalytic efficiency of enzyme activity. It is proposed that the rigidity in protein structure causes the lack of correct vicinity and positioning of the key amino acids required for an efficient catalysis. In the other word, a somewhat flexible in protein architecture is required for binding and accommodating the substrate with the active site properly, and of course for releasing the enzymatic reaction product or products. The structural factors including enhanced number of salt bridges, high hydrophobicity of protein core, longer chain loops are the factors of low flexibility of the thermophilic proteins and also a higher content of proline residue in protein loops seems to correlate with enhanced thermostability in thermophilic proteins^21,33,43–45^, and rSAM has 16 proline residues. Nevertheless, according to the structural studies, it is expected that the rSAM native structure should be at pH 7 and at pH 8 was partially unfolded to occur the interaction between enzyme and substrate well. Investigating the structural changes of the enzyme at different temperatures by intrinsic and extrinsic fluorescence reveals that rSAM has the maximum structural stability at 30 and 40 °C and at optimum temperature (50 °C) has no initial compaction and at 60 °C there is very little difference in protein structure compared to the 50 °C. As a result, rSAM structure exhibits good stability up to 60 °C and this must be related to the rSAM temperature stability. This issue and the protein structural stability at pHs 4–11 approves the rSAM rigidity. Effect of BME as a disulfide bonds reducing agent on the enzyme structure by intrinsic and extrinsic fluorimetry of the enzyme showed that by increasing the concentration of BME, more changes in the rSAM structure would be seen while as we have seen in Table 1, at the high BME concentration the rSAM activity was more than that of in the presence of the low BME concentration. This result is similar to the previous ones confirms that the rSAM possesses rigid structure and with the relative unfolding under various conditions could have better performance to interact with the substrate.

Study of the effect of acrylamide as a quencher at pHs 4, 8 and 11 shows the lowest Stern-Volmer constant at the enzyme optimum pH (8). This result suggests that at pH 8 tryptophan residue is less exposed to the solvent than the pHs 4 and 11. Acrylamide quenching research at different temperatures shows that at optimum temperature (50 °C) tryptophan residue is more exposed to the solvent compare to the 30 and 40 °C which is indicating a relative decrease in the rSAM structural stiffness under its optimal conditions. The results of acrylamide quenching effect on the rSAM in presence of the 5, 10 and 30 mM BME final concentration are revealing the effect of higher BME concentration on the further changing the rSAM structure and with regard to the more rSAM activity at the higher BME concentration we can draw the conclusion and make sure that the rSAM has a constricted structure and when its structure goes out of the natural state can convert L-glutamine to L-glutamate more efficiently.

Circular dichroism spectroscopy is broadly used to evaluate the alterations in the secondary structure of proteins and their folding properties^46^. Since the resulting CD spectra of rSAM did not show many changes in the rSAM secondary structure and contents under pHs 4, 8, 11, temperatures 40, 50 and 60 °C, and the presence of BME, it can explain the rSAM relative stability under the above conditions. A slight change in rSAM secondary structure despite its increasing activity by enhancing BME concentration, confirms that the BME by reducing the disulfide bond(s) and increasing the flexibility of the rSAM tertiary structure causes more activity by it and there is not much impression on the rSAM secondary structure by BME.

As is clear in Supplementary Fig. S6,A,the presence of cysteine (Cys 203) in the enzyme active site is in agree with the enzyme reducing activity in the presence of iodoacetate and iodoacetamide as thiol binding agents. A disulfide bond in the rSAM predicted 3D structure (Supplementary Fig. S6,B) confirms its activity and structural changing by the DTT and BME as the disulfide bond reducing agents.

## Materials and Methods

### Chemicals, bacterial strains, plasmid and culture conditions

*Pfu* DNA polymerase, RNase A, *Nde* I and *Not* I, T4 DNA ligase, α-chymotrypsin, pepsin, trypsin, proteinase K and DNA ladder were purchased from Fermentase. Nessler reagent (CAS Number: 7783-33-7), kanamycin (K1377–5G), agarose (A9539–50G) ANS (A 1028) were provided from sigma Aldrich Co (Steinheim, USA). Anti poly-Histidine-HRP antibody was purchased from sigma (A7058). Protein molecular mass marker, IPTG, amino acids, DTT, BME, GSH, ascorbic acid, iodoacetate, iodoacetamide were acquired from Merck. DNA extraction kit was purchased from Peqlab and PCR product purification kit was obtained from BioNEER (Seoul, Korea). Ni-NTA resin was purchased from Invitrogen (Carlsbad, USA). *E. coli* DH5α and BL21 (DE3) strains and pET-26b vector resistant to kanamycin were purchased from Invitrogen (Carlsbad, USA). All experiments were replicated at least three times.

Cultivation of *Cohnella* sp. A01, was carried out at 55 °C in LB medium for 4 days and it΄s genomic DNA was extracted using the high pure DNA extraction kit. *E. coli* strains containing pET-26b vector were cultured at 37 °C in LB with final concentration 30 μg/ml of kanamycin. LB plates were solidified with 1.5% agar in the presence and absence of kanamycin to perform respectively contamination and viability tests.

### Molecular cloning, expression, purification and Western blotting of L-glutaminase

SAM´s gene (Coh 03501.n) was PCR amplified with a pair of gene specific primers comprising appropriately engineered restriction sites. Forward primer has *Nde*I recognition site (underlined) (5′GGGAATTCCATATGCCTTCGGACGACATCG3′) (Tm = 59.5 °C) and the reverse primer has *Not* I recognition site (underlined) (5′AAGGAAAAAAGCGGCCGCAAACATGCTCCAGTCGAATTC3′) (57.5 °C). The number of bases flanking the recognition sequences are shown in red. PCR amplicon was purified using the PCR purification kit and the amplified and purified DNA segment and also pET-26b were simultaneously digested by restriction enzymes (*Nde* I, *Not* I). Ligation of the restricted fragment with a size of 945 bp and pET-26b was then carried out by using the T4 DNA ligase and the recombinant plasmid was transformed into the competent *E. coli* DH5α.

rpET-26b transformed into *E. coli* BL21 (DE3) and 2 mL of the overnight transformed grown cells, were inoculated in 50 mL LB containing 50 μl kanamycin (30 mg/mL) and incubated at 37 °C. IPTG at a final concentration of 1 mM was used to induce overexpression of protein in recombinant cells and incubated at 27 °C for 16 h with 120 rpm. Subsequently, recombinant cells were harvested by centrifugation at 8000 g for 20 min at 4 °C. The resuspension of rpET-26b carrying cells in 4 mL ice-cold lysis buffer containing 50 mM NaH_2_PO_4_, 300 mM NaCl, 10 mM imidazole and 0.05% tween 20 (pH 8) was done and followed by sonication. Centrifugation of cell extracts at 8000 g for 45 min at 4 °C was carried out and the purification of the supernatant by using Ni-NTA Sepharose affinity chromatography at 4 °C was done. Purified rSAM was dialyzed against 50 mM phosphate buffer at pH 8 and run and tracked on 12.5% SDS-PAGE and the molecular weight of rSAM according to protein molecular mass marker was analyzed using SDS-PAGE.

rSAM expression was also confirmed using Western blot analysis according to the His-tag detection by using conjugated anti poly-Histidine-HRP antibody against expressed histidine sequence tag of the recombinant protein on PVDF membrane in the presence of DAB as substrate.

### Qualitative and quantitative L-glutaminase assay

A rapid plate assay to detect the rSAM activity was performed on agar medium containing L-glutamine and phenol red as respectively enzyme substrate and pH indicator. 100 μl of purified rSAM dialyzed in pH 7 was poured into a well with 8 mm diameter at the center of the medium and incubated at 50 °C for 1 hour. Formation of pink colored zone around the well against the yellow background was compared to the control wells. One of the control wells contains phosphate buffer with pH 7 in which enzyme was dialyzed and another one contains water.

rSAM quantitative activity was measured using Nesslerization reaction according to the NH_3_ liberating from specific substrate of the enzyme (L-glutamine) as described by Imada *et al*. with a slight modification^47^. The mixture reaction containing an aliquot of 20 μl of enzyme preparation (0.4 mg/ml) with 20 μl of 40 mM L-glutamine solution and 20 μl of 50 mM phosphate buffer (pH 8) was incubated at 50 °C for 15 min. All aliquots were pre-warmed at 50 °C. Termination of the reaction was occurred by the addition of 20 μl of 2 N acetic acid. After 10 min 40 μl Nessler reagent and 740 μl distilled water were added to the 20 μl of the mixture and after 5 min, liberated ammonia was quantified using spectrophotometer at 450 nm against the mixture without enzyme as control and rSAM unit was determined basis on the ammonium sulphate previously prepared curve as standard. One glutaminase unit (IU) is defined as the amount of enzyme that liberates 1 μmol ammonia per minute under the standard assay conditions. Protein concentration was determined via Bradford΄s method and BSA was used as the standard solution.

### Substrate specificity

In order to determine the rSAM substrate specificity, some molecules containing amide bond including L-Asparagine, Urea and Acrylamide at the concentration of 40 mM were separately added to the assay mixture in place of L-glutamine and enzyme assay was conducted under the standard conditions. Results were expressed as the percentage of the enzyme relative activity in the presence of various substrates against the enzyme activity with L-glutamine.

### Effect of temperature and pH on the rSAM activity and stability

rSAM temperature profile was determined by carrying out the enzyme assay conditions as previously described over a temperature range of 10 to 90 °C. To assess the enzyme stability in different temperatures, rSAM activity was measured after its incubation for 90 min in temperatures ranging 10 to 90 °C and thermostability of the enzyme was also estimated by incubation of it at 40, 50, 60 & 70 °C for 6 h and the enzyme activity was measured every 1 h. In order to study the rSAM half-life at room temperature, enzyme was incubated at 25 °C for 30 days and its activity was measured every two days.

To estimate the effect of pH on the rSAM activity, enzyme was dialyzed against 5 mM phosphate buffer at pH 7 and 100 mM mixed buffer composed of acetate, phosphate and glycine which was adjusted to the various pHs was used to determine the enzyme activity at different pHs. To assess the L-glutaminase pH stability, enzyme was preincubated in 5 mM mixed buffer at various pHs (3–12) for 90 min and the enzyme activity was then determined in 100 mM phosphate buffer (pH 8) and also the rSAM pH stability was assessed by preincubation it in 5 mM mixed buffer at pH 5, 8 and 11 for 6 h and the measurement its activity in optimum conditions was done every hour.

### The influence of various reagents and effectors on the rSAM activity

To investigate the enzyme activity in the presence of different metal ions, reducing and thiol binding agents, chemical solvents, chelator and detergents, purified rSAM was incubated to each factor with a certain concentration separately for 30 min and the enzyme activity was reported as relative activity compare to the untreated enzyme.

### rSAM salt tolerance

The stability of purified rSAM at various concentrations of NaCl (0–25% w/v) for 90 min was assayed in the standard reaction mixture and the specific activity was calculated at rSAM optimum conditions.

### rSAM resistance against proteases

The stability of the rSAM to proteases including chymotrypsin, pepsin, trypsin and proteinase K was investigated by incubation of the rSAM and each protease for 15 min at 37 °C at a protease: substrate ratio of 1:20 v/v. BSA was used as control. Pepsin´s activity depends on the environment pH and the maximum activity of this proteolytic enzyme is exhibited at pH 2 and inactivated at pH 8 and above. So to get the best result for pepsin digestion, before incubation of the rSAM and pepsin, rSAM was dialyzed at pH 7 and 1 N HCl was added to a final concentration of 0.04 N. Digestion reactions were analyzed by SDS-PAGE.

### rSAM Kinetic and thermodynamic parameters

rSAM Michaelis-Menten constant (K_m_), Maximum velocity (V_max_) values were determined in the presence of the natural substrate L-glutamine with a concentration range of 0–25 mM and MgCl_2_. Michaelis-Menten plot was applied to calculate the K_m_ and V_max_ in GraphPad Prism 6 software. Catalytic efficiency was calculated as kcat/Km.

Based on the temperature profile up to the 50 °C (the rSAM optimum temperature) and the Arrhenius plot slope, rSAM thermo-activation parameters for substrate to product conversion (ΔE^*^, ΔH^*^, ΔG^*^, ΔS^*^) were calculated.

To determine the irreversible thermo-inactivation parameters, according to the temperature stability and this issue that the enzyme activity over the time reduces log linearly:

$$\mathrm{ln}({A}_{t}/{A}_{0})=\mbox{--}kt,$$

A_t_ is the enzyme activity at time t, A_0_ is the initial enzyme activity, t is the treatment time, and k is the first-order inactivation rate constant, the rate constant dependence to the temperature for the enzyme inactivation was analyzed based on the Arrhenius plot and the Arrhenius plot slope was used to obtain the activation energy for the heat inactivation (Δ*E*^#^). Activation enthalpy (Δ*H*^#^), and free energy of the inactivation (Δ*G*^#^) and the activation entropy (ΔS^#^) for the rSAM heat inactivation were also calculated.

The rate constant of the rSAM inactivation at temperatures 40, 50, 60 and 70 °C was used to the rSAM half-life (t_1/2_) estimation as follows:

$${{\rm{t}}}_{1/2}=\,\mathrm{ln}(2)/{{\rm{k}}}_{{\rm{in}}}$$

### Structural studies

#### Fluorescence analysis

Fluorescence detections were carried out by using a kary eclipse luminescence spectrometer and slit width of both excitation and emission was set at 10 nm. rSAM tryptophan exposure and ANS binding to its exposed hydrophobic surfaces at different pHs (3–12), temperatures (30–100 °C) and in the presence of final concentrations 5, 10 & 30 mM of BME was surveyed. After 90 min pre-incubation of protein solution with a final concentration 0.25 mg/ml at pHs 3–12 (at room temperature), temperatures 30–100 °C and 30 min pre-incubation in the presence of the 5, 10 & 30 mM of BME at room temperature separately, intrinsic spectra were measured at wavelengths between 300–400 nm with the excitation wavelength of 280 nm.

ANS binding to the rSAM exposed hydrophobic surfaces was checked out after mentioned incubations of the same samples and extrinsic spectra were recorded between 400 to 600 nm with the excitation wavelength of 360 nm following the adding 2 µL of a 10 mM ANS preparation dissolved in 1 M NaOH to the 398 µL sample and incubated for 10 min at 25 °C. A sample without protein was also used as control.

Quenching measurements using acrylamide as a quencher of the solvent-exposed tryptophan as a fluorophore described by Eftink and Ghiron^48^. In order to determine the most acceptable concentration range of the acrylamide as a quencher to assess the tryptophan solvent exposure, fluorescence quenching was done by firstly incubating the rSAM solutions separately at pHs 3, 8 &11 at 25 °C and temperatures from 30 to 70 °C for 90 min and in the presence of final concentrations 5, 10 & 30 mM of BME for 30 min at 25 °C and subsequently was followed by adding the different volumes of the 2 M acrylamide stock solution to the incubated rSAM with final concentration 0.25 mg/ml. Each sample was incubated for 30 min and then measurement with the aim of titration was carried out at the wavelength range of 300–400 nm with the adjusted excitation wavelength at 280 nm. Depends on the collision between quencher and fluorophore, the ratio of F0/F against acrylamide concentration[Q]was plotted to acquire the quenching of rSAM.

#### Circular dichroism analysis

To elucidate the secondary structure of the rSAM under specific conditions, rSAM was assessed using Far-UV CD spectroscopy on an Aviv spectropolarimeter Model-215 (USA). Enzyme was dialyzed against 5 mM phosphate buffer at pH 7. CD spectra monitoring at enzyme concentration of 0.5 mg/ml was performed at temperatures 40, 50 and 60 °C, pHs 4, 8, 11 and in the presence of final concentrations 5, 10 & 30 mM of BME. The Quartz cell path length used for Far- UV CD spectra were 1 mm and the alterations in rSAM secondary structure were measured between 200–250 nm. Molar ellipticity, [Θ] (deg cm^2^dmol^−1^) which is calculated from the following formula, was used to represent the results.

$$[{\Theta }]\lambda =(\theta \times {1000}MRW)/(cl)$$

The measured ellipticity which is in degrees at wavelength λ expresses as θ, MRW is the mean amino acid residue weight, c is the protein concentration in mg/ml and l is the light path length in centimeters. The software by which the rSAM secondary structure was determined is CDNN 2.1.

### *In-silico* analysis

Homology search was performed against NCBI database using the basic local alignment search tool (BLASTp.2.8.1). Multiple sequence alignment and phylogenetic tree were established using respectively clustalW and neighbor-joining method in MEGA7^49^. rSAM bio-molecular 3D structure modeling was done using Modeller 9.18^50^.

## Supplementary information

Supplementary Information

Supplementary Information

Supplementary Information

Supplementary Information

Supplementary Information

Supplementary Information

Supplementary Information

Supplementary Information
