# Supplementary figures and images for: Novel halo- and thermo-tolerant Cohnella sp. A01 L-glutaminase: heterologous expression and biochemical characterization
Source: Sci Rep. 2019 Dec 13;9:19062. doi: 10.1038/s41598-019-55587-9 (PMC6910923; doi:10.1038/s41598-019-55587-9)

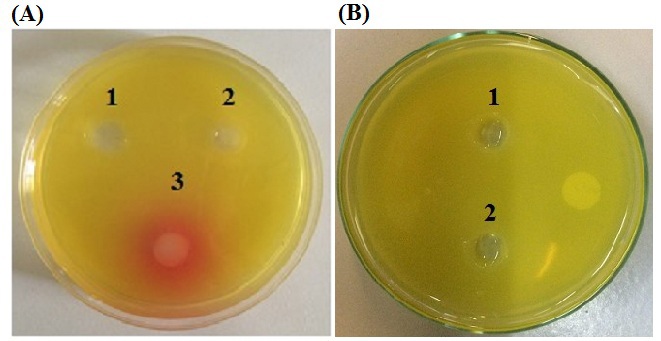

Supplement: Supplementary file 1 — Supplementary Information [file 41598_2019_55587_MOESM1_ESM.jpg]

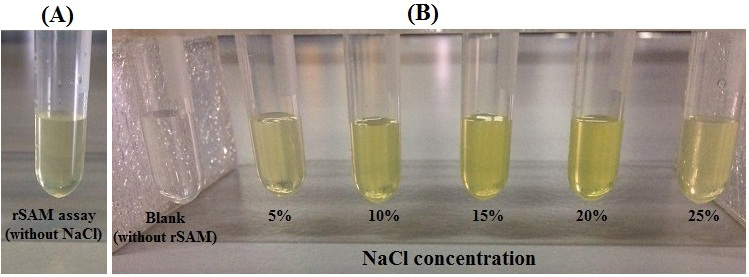

Supplement: Supplementary file 2 — Supplementary Information [file 41598_2019_55587_MOESM2_ESM.jpg]

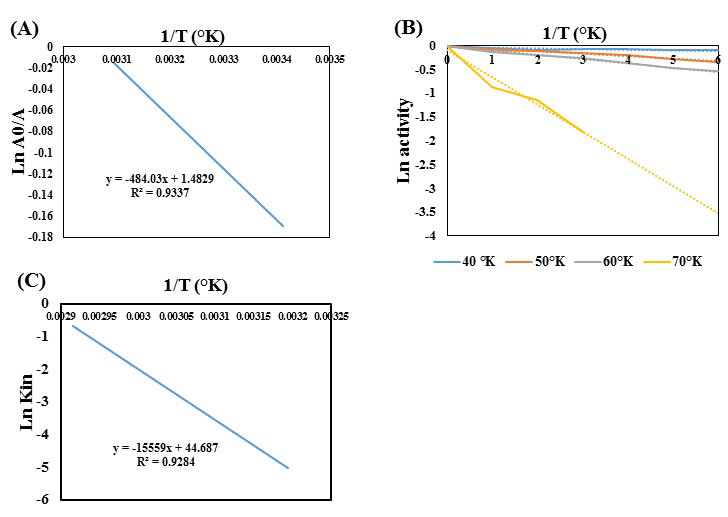

Supplement: Supplementary file 3 — Supplementary Information [file 41598_2019_55587_MOESM3_ESM.jpg]

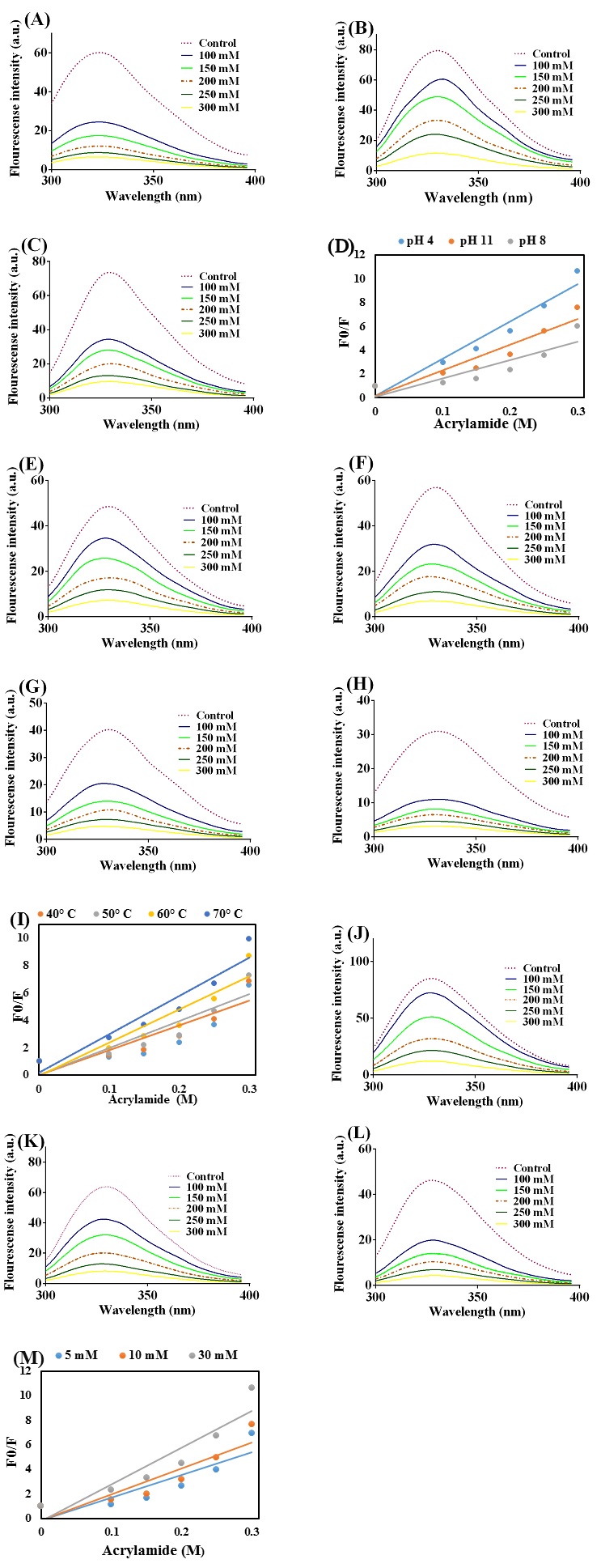

Supplement: Supplementary file 4 — Supplementary Information [file 41598_2019_55587_MOESM4_ESM.jpg]

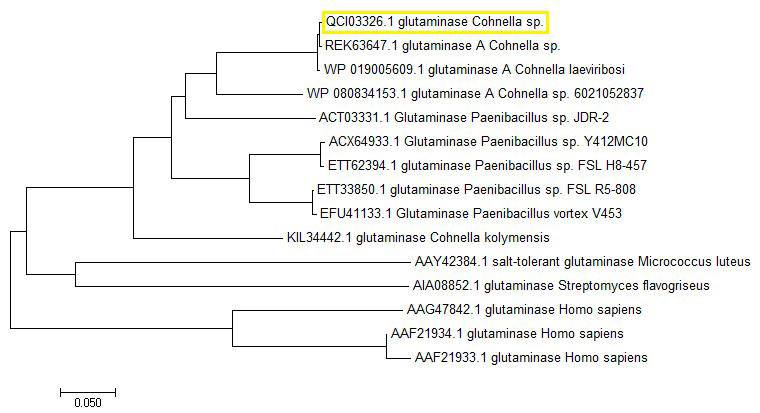

Supplement: Supplementary file 5 — Supplementary Information [file 41598_2019_55587_MOESM5_ESM.jpg]

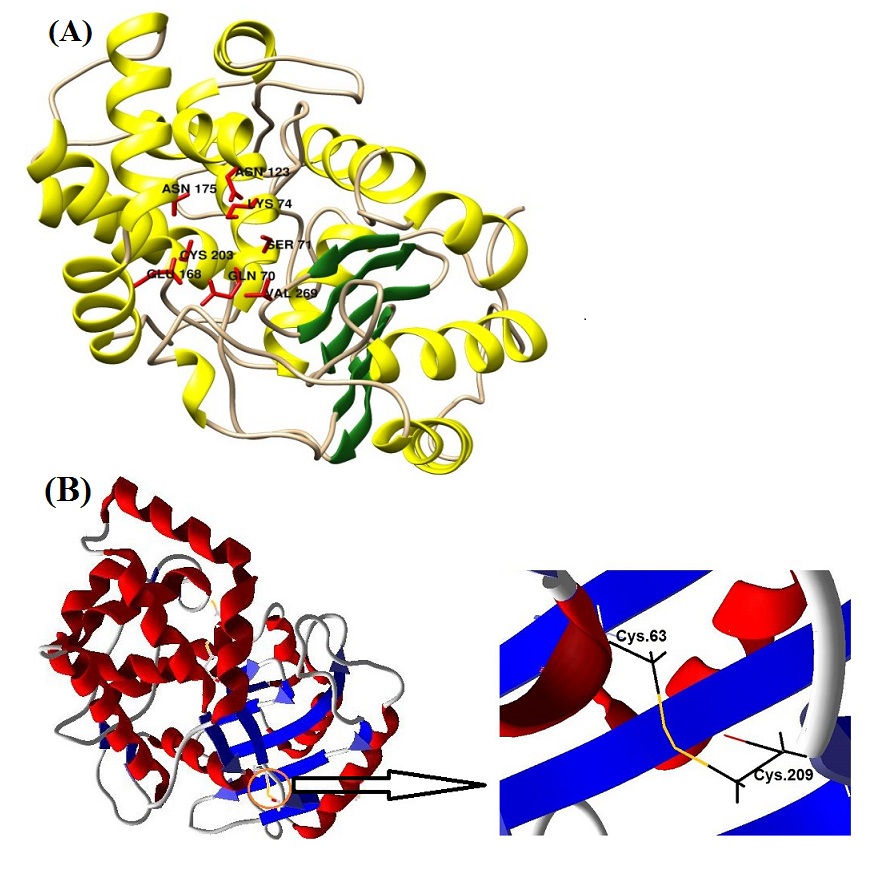

Supplement: Supplementary file 6 — Supplementary Information [file 41598_2019_55587_MOESM6_ESM.jpg]

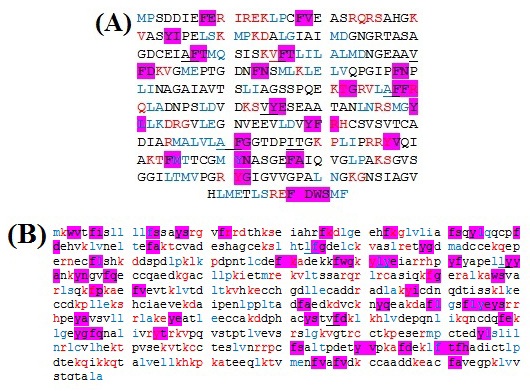

Supplement: Supplementary file 7 — Supplementary Information [file 41598_2019_55587_MOESM7_ESM.jpg]
