# Supplementary material for: Novel halo- and thermo-tolerant Cohnella sp. A01 L-glutaminase: heterologous expression and biochemical characterization
Source: Sci Rep. 2019 Dec 13;9:19062. doi: 10.1038/s41598-019-55587-9 (PMC6910923; doi:10.1038/s41598-019-55587-9)
Supplement: Supplementary file 8 — Supplementary Information [file 41598_2019_55587_MOESM8_ESM.docx]

**Novel halo- and thermo-tolerant *Cohnella* sp. A01 L-glutaminase: heterologous expression and biochemical characterization**

**Samaneh Mosallatpour^1,2^, Saeed Aminzadeh*^1^, Mehdi Shamsara*^3^, Reza Hajihosseini^2^**

^1^ Bioprocess Engineering Group, Institute of Industrial and Environmental Biotechnology, National Institute of Genetic Engineering and Biotechnology (NIGEB), Tehran, Iran

^2^ Faculty of Science, Payame Noor University, Tehran, Iran

^3^ Animal Biotechnology Department, Institute of Agricultural Biotechnology, National Institute of Genetic Engineering and Biotechnology (NIGEB), Tehran, Iran

^*^**Corresponding authors:** Saeed Aminzadeh ([aminzade@nigeb.ac.ir](mailto:aminzade@nigeb.ac.ir)), Mehdi Shamsara (shamsa@nigeb.ac.ir)

Tel: +98 21 44787412; Fax: +98 21 44787399

**Supplementary Information**

| **Supplementary Table S1. rSAM thermodynamic parameters at optimum temperature (50^°^ C) and its half-life at various temperatures.** | | | | | | | | | | | |
| --- | --- | --- | --- | --- | --- | --- | --- | --- | --- | --- | --- |
| Irreversible thermo-inactivation parameters | | | | Thermo-activation parameters | | | | rSAM Half-life (h) | | | |
| ΔE^#^  (kJ/mol) | ΔG^#^  (kJ/mol) | ΔH^#^  (kJ/mol) | ΔS^#^  (kJ/mol^°^K) | ΔE^*^  (kJ/mol) | ΔG^*^  (kJ/mol) | ΔH^*^  (kJ/mol) | ΔS^*^  (kJ/mol^°^K) | 40  (°C) | 50  (°C) | 60  (°C) | 70  (°C) |
| 105 | 58 | 102 | 0.13 | 4 | 58 | 1.3 | -0.17 | 52.1 | 13.2 | 7.8 | 1.2 |

| **Supplementary Table S2. rSAM´s Stern-Volmer constants (Ksv) at different pHs, temperatures and BME concentrations.** | | | | | |
| --- | --- | --- | --- | --- | --- |
| Stern-Volmer constant (M^-1^) | BME (mM) | Stern-Volmer constant (M^-1^) | Temperature (°C) | Stern-Volmer constant (M^-1^) | pH |
| 19.9 | 5 | 18.7 | 30 | 28.1 | 4 |
| 22.1 | 10 | 19.7 | 40 | 16.8 | 8 |
| 32.1 | 30 | 20 | 50 | 22.1 | 11 |
| - | - | 25.8 | 60 | - | - |
|  | - | 30 | 70 | - | - |

| **Supplementary Table S3. SAM´s secondary structure contents at optimum temperature and pH.** | | | | | |
| --- | --- | --- | --- | --- | --- |
| Secondary structure | Helix  (%) | Antiparallel (%) | Parallel  (%) | Beta-Turn  (%) | Random. Coil (%) |
| rSAM at 50 °C | 9.7 | 16.8 | 14.4 | 14.4 | 44.5 |
| rSAM at pH 8 | 9.6 | 17 | 14.5 | 14.3 | 44.8 |

| **Supplementary Table S4. Comparison of some features of L-glutaminases from various microorganisms.** | | | | | | | |
| --- | --- | --- | --- | --- | --- | --- | --- |
| Microorganism | Molecular weight (kDa) | Optimum temp (°C) | Optimum pH | NaCl tolerance | Substrate (K_m_ or activity) | K_m_ (mM) | Ref |
| *Cohnella* sp. A01  (This study, rSAM) | 34 | 50 | 8 | 95% of enzyme activity at 25% NaCl | L-Gln(100%)  No activity toward other tested molecules | 1.8 | - |
| *Streptomyces pratensis* NRC 10 | 46 | 45 | 9 | 10% of enzyme activity at 20% NaCl | L-Gln(100%)  D-Gln(35%) | 0.175 | ^3^ |
| Bacillus sp. LKG-01 (MTCC 10401) | 66 | 70 | 11 | 80% of enzyme activity at 25%(*w/v*) NaCl | L-Gln(100%  D-Gln(18%)  L-Asn(02%)  D-Asn(08%)  L-Gln-L-Asn(90%)  L-Asn–D-Asn(24%)  D-Gln–L-Asn(48%)  N-Acetyl–L-Asn(5.1%) | 0.240 | ^4^ |
| *Streptomyces avermitilis* | 50 | 30 | 7, 8 | 4% NaCl | - | - | ^5^ |
| *Aspergillus oryzae* NRRL32567 | 68 | 30-40 | 7 | 60% of enzyme activity at 20%(*w/v*) NaCl | L-Gln(100%)  D-Gln(23%)  L-Asn(18%)  D-Asn(10%) | 4.5 | ^6^ |
| *Aspergillus oryzaezae* AJ11728 | 82 | 37-45 | 9 | - | L-Gln(100%)  D-Gln(106%) | 1.2 | ^7^ |
| *Acinetobacter glutaminasificans* | 33 | - | 7 | - | L-Gln(5.8µM)  D-Gln(1.9 µM)  L-Asn(4.8 µM)  D-Asn(1.6 µM) | 0.0058 | ^8,9^ |
| *Pseudomonas aeruginosa, (GLS A, GLS B)* | 137  67 | - | 7.5-9 | - | L-Gln(0.12mM)  D-Gln(0.85mM)  L-Asn(0.068mM)  D-Asn(0.105mM)  L-Gln(0.18mM)  D-Gln(0.23mM)  L-Theanine(0.71mM)  D-Theanine(0.92mM) | 0.11  0.18 | ^10,11^ |
| *Brevundimonas diminuta* MTCC 8486 | 140 | - | - | 87.5% of enzyme activity at 4%(*w/v*) NaCl | - | - | ^12^ |
| *Bacillus cereus* MTCC 1305 | 35 | 35 | 7.5 | - | L-Gln(100%)  D-Gln(48.4%)  L-Glu(13.56%) | 6.25 | ^13^ |
| *Lactobacillus reuteri* KCTC3594 | 70 | 40 | 7.5 | 50% of enzyme activity at 20%(*w/v*) NaCl | - | 4.5 | ^14^ |
| *Aspergillus sojae* | 110 | 50 | 9 | 5% of enzyme activity at 18% NaCl | L-Gln(720μmol.min^−1^ mg^−1^)  D-Gln(50.6)  Cbz-Gln(32.4)  L-Ala-Gln(236.2)  L-Gly-Gln(308.3)  L-Leu-Gly-Gln(16.3)  L-Pro-Leu-Gly-Gln (73.2)  Cbz-Gln-Gly(29.9)  L-Asn(293.7)  L-Gly-Asn(21.9)  L-Leu-Ala-Asn(4.8)  L-Leu-Gly-Asn(20.8)  L-Pro-Leu-Gly-Asn (36.5) | 9 | ^15^ |
| *Stenotrophomonas*  *maltophilia* NYW-81 | 36 | 60 | 9 | 86% of enzyme activity at 16% NaCl | L-Gln(100%)  D-Gln(86%)  L-Asn(73%)  D-Asn(68%) | - | ^16^ |
| *Streptomyces canarius* FR (KC460654) | 44 | 40 | 8 | 40% of enzyme activity at 25% NaCl | L-Gln(0.129mM)  L-Asn(0.137mM)  L-Asp(5.38mM) | 0.129 | ^17^ |
| *Streptomyces sp* | 50 | 30 | 7 | 29.7% of enzyme activity at 5%(*w/v*) NaCl | - | 2.8 | ^18^ |
| *Bacillus pasteurii* | 55 | 37 | 9 | 4.9 | L-Gln(100%)  D-Gln(8.1%)  L-Asn(20.6%)  D-Asn(<0.1%) | 9.5 | ^19^ |
| *Micrococcus luteus* K-3(GLS I, GLS II) | 86 (48 , 38),  86 | 50,  50 | 8,  8.5 | 100% activity at 8-16%,  80% activity at 8-16% NaCl | L-Gln(100%)  L-Glu ɤ-methylester(38%)  L-Glu ɤ-ethylester(9%)  L-Glu ɤ-benzylester(2%)  L-Glu ɤ-hydroxamate(4%)  ɤ-L-Glutamyl L-Glu(2%)  L-Gln(100%)  L-Glu ɤ-methylester(27%)  L-Glu ɤ-ethylester(8%)  ɤ-L-Glutamyl L-Glu(2%) | 4.4, 6.5 | ^20^, ^21^ |
| Aspergillus oryzae RIB40 (AoGls) | 49.9 | 30 | 8-9 | 54% of enzyme activity at 2.9M NaCl | - | 4.5 | ^22^ |
| *Bucillus subtilis:* YbgJ, Yb1M | 36.2,  34 | - | 7.5-8,  7.5-8 | - | L-Gln(100%)  D-Gln(No activity)  L-Asn(No activity)  L-Gln(100%)  D-Gln(No activity)  L-Asn(No activity) | 30.6, 7.6 | ^23^ |
| *E. coli* YneH, YbaS | 33.5  32.9 | - | 7.5-8  7.5-8 | -  Not effect up to 0.2M NaCl | L-Gln(100%)  D-Gln(No activity)  L-Asn(No activity)  L-Gln(100%)  D-Gln(No activity)  L-Asn(No activity) | 27.6, 7.3 | ^23^ |
| *Bacillus amyloliquefaciens* | 35 | 60 | 6.5 | 68% of enzyme activity at 20% NaCl | - | - | ^24^ |
| *Peudomonas acidovorance* | 39 | - | 9.5 | - | L-Asn(100%)  D-Asn(50%)  L-Gln(147%)  D-Gln(58%)  L-Asn + D-Asn(99%)  L-Asn + L-Gln(110%)  L-Asn + D-Gln(74%)  L-Asp-β-hydroxamate(91%)  D-Asp-β-hydroxamate(29%)  L-Glu-ɤ-monohydroxamate(98%)  N-Acetyl-L-asparagine(5.2%) | 0.022 | ^25^ |
| *Pseudomonas nitroreducens* IFO 12694 | 40 | - | 9 | - | L-Gln(100%)  D-Gln(107%)  ɤ-Glutamyl nitroanilide(85%)  ɤ-Glutamyl methylamide(78%)  Theanine(74%)  Glutathione(82%) | 6.5 | ^26^ |
| *Rhizobium etli* | 106.8 | 45 | 8.3-8.5 | - | - | 1.5 | ^27^ |
| *Debaryomyces spp* | 50.65 | 40 | 8.5 | - | l-Gln(100%)  l-g-Glu-methyl ester(48.7%)  CBZ-l-Gln-Gly(0.2%)  l-Albizziin(9.2%) | 4.5 | ^28^ |
| *Lactobacillus rhamnosus* | - | 50 | 7 | 90% of enzyme activity at 20%w/v NaCl | - | 4.8 | ^29^ |
| *Meyerozyma (Pichia) guilliermondii* EM2Y61 | - | 30 | 4.5 | 0.43 U/mg at 18%w/v NaCl | - | - | ^30^ |

**Legends of the Supplementary Figures**

**Supplementary Figure S1.** (A) The Agar-phenol red plate contains L-glutamine: (1) Water-containing well. (2) The well containing buffer in which enzyme has been dialyzed. (3) Red halo around the enzyme-containing well indicates ammonia liberation from L-glutamine by rSAM. (B) The Agar-phenol red plate without L-glutamine: (1) The well containing buffer in which enzyme has been dialyzed. (2) The enzyme-containing well without any color change.

**Supplementary Figure S2. rSAM NaCl tolerance assays.** (A) rSAM assay at its optimum pH (8) and temperature (50 ^°^C): the yellow color has been resulted in the interaction of ammonia produced from L-glutamine with the Nessler reagent. (B) By increasing the NaCl concentration up to 20 %, rSAM activity was enhanced and 25 % salinity level reduced the enzyme activity less than only 5%.

**Supplementary Figure S3. rSAM thermodynamic parameters.** (A) Arrhenius plot for rSAM temperature activation up to 50 ^°^C, The activation energy was 4 kJ/mol. (B) Heat inactivation kinetics at temperatures 40-70 ^°^C. (C) rSAM irreversible heat inactivation Arrhenius plot. The activation energy for thermo- inactivation was 105 kJ/mol. Each value represents the mean ± SD for three determinations. The software used was excel 2013, <https://www.microsoft.com/en-us/download/office.aspx>.

**Supplementary Figure S4. rSAM fluorescence emission spectra in the presence of increasing concentration of acrylamide.** At pHs (A) 4, (B) 8, (C) 11 and (D) Stern-Volmer plots corresponding to tested pHs: acrylamide at the rSAM´s optimum pH (8) showed the least quenching effect on the exposed tryptophan residue compare to the pHs 4 and 11. At temperatures (E) 40, (F) 50, (G) 60, (H) 70 and (I) Stern-Volmer plots corresponding to tested temperatures: at optimum temperature (50 ^°^C) acrylamide quenched the tryptophan residue more than at 40 ^°^C. At BME final concentrations of (J) 5, (K) 10, (L) 30 mM, (M) corresponding Stern-Volmer plot: at 30 mM final concentrations of BME tryptophan residue was more quenched by acrylamide than at 5 and 10 mM. The software used was “GraphPad prism 6”, <https://www.graphpad.com/scientific-software/prism/>.

**Supplementary Figure S5.** Phylogenetic tree of the rSAM amino acid sequence by glutaminases from the some other microorganisms and homo sapiens. The software used was “MEGA7.0.14^1^”, <https://www.megasoftware.net/>.

**Supplementary Figure S6.** (A) rSAM 3D structure modeling and putative active site amino acids: Gln70, Ser71, Asn123, Glu168, Asn175, Cys203, Val269, Lys74. (B) Modeled disulfide bond in rSAM 3D structure. The software used was “Modeller 9.18” ^2^, <https://salilab.org/modeller/>.

**Supplementary Figure S7.** Cleavage sites of Chymotrypsin (purple high lighted), pepsin (underlined), trypsin (red color) and proteinase K (has very much cleavage sites so not shown) on the rSAM (A) and BSA (B) amino acid sequences.

**References:**

1 Hall, B. G. Building phylogenetic trees from molecular data with MEGA. *Molecular biology and evolution* **30**, 1229-1235, doi:10.1093/molbev/mst012 (2013).

2 Zhang, Y. I-TASSER server for protein 3D structure prediction. *BMC bioinformatics* **9**, 40, doi:10.1186/1471-2105-9-40 (2008).

3 Tork, S. E., Aly, M. M. & Elsemin, O. A new l-glutaminase from Streptomyces pratensis NRC 10: Gene identification, enzyme purification, and characterization. *Int J Biol Macromol* **113**, 550-557, doi:10.1016/j.ijbiomac.2018.02.080 (2018).

4 Kumar, L., Singh, B., Adhikari, D. K., Mukherjee, J. & Ghosh, D. A temperature and salt-tolerant L-glutaminase from gangotri region of uttarakhand himalaya: enzyme purification and characterization. *Applied biochemistry and biotechnology* **166**, 1723-1735, doi:10.1007/s12010-012-9576-0 (2012).

5 Amer, S., Khalil Habeeb, M. & Ahmed Abdallah, N. *Production, purification and characterization of L- glutaminase enzyme from Streptomyces avermitilis*. Vol. Vol. 7 (2013).

6 Bazaraa, W., Alian, A., El-Shimi, N. & Mohamed, R. Purification and characterization of extracellular glutaminase from Aspergillus oryzae NRRL 32567. *Biocatalysis and Agricultural Biotechnology* **6**, 76-81, doi:<https://doi.org/10.1016/j.bcab.2016.02.009> (2016).

7 Koibuchi, K., Nagasaki, H., Yuasa, A., Kataoka, J. & Kitamoto, K. Molecular cloning and characterization of a gene encoding glutaminase from Aspergillus oryzae. *Applied microbiology and biotechnology* **54**, 59-68 (2000).

8 Roberts, J., Holcenberg, J. S. & Dolowy, W. C. Isolation, crystallization, and properties of Achromobacteraceae glutaminase-asparaginase with antitumor activity. *The Journal of biological chemistry* **247**, 84-90 (1972).

9 Nandakumar, R., Yoshimune, K., Wakayama, M. & Moriguchi, M. Microbial glutaminase: biochemistry, molecular approaches and applications in the food industry. *Journal of Molecular Catalysis B: Enzymatic* **23**, 87-100, doi:<https://doi.org/10.1016/S1381-1177(03)00075-4> (2003).

10 Soda, K., Oshima, M. & Yamamoto, T. Purification and properties of isozymes of glutaminase from Pseudomonas aeruginosa. *Biochem Biophys Res Commun* **46**, 1278-1284 (1972).

11 Ohshima, M., Yamamoto, T. & Soda, K. *Further Characterization of Glutaminase Isozymes from Pseudomonas aeruginosa*. Vol. 40 (1976).

12 Jayabalan, R., Jeeva,S.,Sasikumar,A.P.,Inbakandan,D.,Swaminathan,K.,Yun,S.E.,. Extracellular L-glutaminase productionbymarine Brevundimonas dimin-

uta MTCC 8486. *Int.J.Appl.Bioeng.* **3**, 4-9 (2011).

13 Singh, P. & Banik, R. M. Biochemical characterization and antitumor study of L-glutaminase from Bacillus cereus MTCC 1305. *Applied biochemistry and biotechnology* **171**, 522-531, doi:10.1007/s12010-013-0371-3 (2013).

14 Jeong, J. M., Lee,H.,Jae-Seong,S. Glutaminase activity of lactobacillus reuteri KCTC3594 and expression of the activity in other lactobacillus spp. by introduction of the glutaminase gene. *Afri.J.Microbiol.Res.* **3**, 605-609 (2010).

15 Ito, K., Hanya, Y. & Koyama, Y. Purification and characterization of a glutaminase enzyme accounting for the majority of glutaminase activity in Aspergillus sojae under solid-state culture. *Applied microbiology and biotechnology* **97**, 8581-8590, doi:10.1007/s00253-013-4693-4 (2013).

16 Wakayama, M. *et al.* Characterization of salt-tolerant glutaminase from Stenotrophomonas maltophilia NYW-81 and its application in Japanese soy sauce fermentation. *Journal of industrial microbiology & biotechnology* **32**, 383-390, doi:10.1007/s10295-005-0257-7 (2005).

17 Reda, F. M. Kinetic properties of Streptomyces canarius L- Glutaminase and its anticancer efficiency. *Brazilian Journal of Microbiology* **46**, 957-968 (2015).

18 Savitha S. Desai, Sonal J. Chopra & Hungund, B. S. Production, purification and characterization of L-Glutaminase from Streptomyces sp. isolated from soil. *Journal of Applied Pharmaceutical Science* **Vol. 6 (07)**, 100-105, doi:10.7324/JAPS.2016.60715 (2016).

19 Klein, M., Kaltwasser, H. & Jahns, T. Isolation of a novel, phosphate-activated glutaminase from Bacillus pasteurii. *FEMS microbiology letters* **206**, 63-67 (2002).

20 Moriguchi, M., Sakai, K., Tateyama, R., Furuta, Y. & Wakayama, M. Isolation and characterization of salt-tolerant glutaminases from marine Micrococcus luteus K-3. *Journal of Fermentation and Bioengineering* **77**, 621-625, doi:<https://doi.org/10.1016/0922-338X(94)90143-0> (1994).

21 Yoshimune, K., Shirakihara, Y., Wakayama, M. & Yumoto, I. Crystal structure of salt-tolerant glutaminase from Micrococcus luteus K-3 in the presence and absence of its product L-glutamate and its activator Tris. *The FEBS journal* **277**, 738-748, doi:10.1111/j.1742-4658.2009.07523.x (2010).

22 Masuo, N. *et al.* Micrococcus luteus K-3-type glutaminase from Aspergillus oryzae RIB40 is salt-tolerant. *Journal of bioscience and bioengineering* **100**, 576-578, doi:10.1263/jbb.100.576 (2005).

23 Brown, G. *et al.* Functional and structural characterization of four glutaminases from Escherichia coli and Bacillus subtilis. *Biochemistry* **47**, 5724-5735, doi:10.1021/bi800097h (2008).

24 Mao Ye, X. L. a. L. Z., 2013. Production of a Novel Salt-tolerant L-glutaminase from Bacillus amyloliquefaciens Using Agro-industrial Residues and its Application in Chinese Soy Sauce Fermentation. *Biotechnology* **12**, 25-35 (2013).

25 Davidson, L., Brear, D. R., Wingard, P., Hawkins, J. & Kitto, G. B. Purification and properties of L-glutaminase-L-asparaginase from Pseudomonas acidovorans. *Journal of Bacteriology* **129**, 1379-1386 (1977).

26 Takashi, T. *et al.* Purification and Some Properties of Glutaminase from Pseudomonas nitroreducens IFO 12694. *Bioscience, biotechnology, and biochemistry* **60**, 1160-1164, doi:10.1271/bbb.60.1160 (1996).

27 Huerta-Saquero, A., Calderon, J., Arreguin, R., Calderon-Flores, A. & Duran, S. Overexpression and purification of Rhizobium etli glutaminase A by recombinant and conventional procedures. *Protein expression and purification* **21**, 432-437, doi:10.1006/prep.2001.1394 (2001).

28 Dura, M. A., Flores, M. & Toldra, F. Purification and characterisation of a glutaminase from Debaryomyces spp. *Int J Food Microbiol* **76**, 117-126 (2002).

29 Weingand-Ziadé, A., Gerber-Décombaz, C. & Affolter, M. Functional characterization of a salt- and thermotolerant glutaminase from Lactobacillus rhamnosus. *Enzyme and Microbial Technology* **32**, 862-867, doi:<https://doi.org/10.1016/S0141-0229(03)00059-0> (2003).

30 Aryuman, P. *et al.* Glutaminase-producing Meyerozyma (Pichia) guilliermondii isolated from Thai soy sauce fermentation. *Int J Food Microbiol* **192**, 7-12, doi:10.1016/j.ijfoodmicro.2014.09.019 (2015).
